# Contextualising Water Use in Residential Settings: A Survey of Non-Intrusive Techniques and Approaches

**DOI:** 10.3390/s16050738

**Published:** 2016-05-20

**Authors:** Davide Carboni, Alex Gluhak, Julie A. McCann, Thomas H. Beach

**Affiliations:** 1 ICRI Sustainable Connected Cities, Intel Corp., London SW7 2AZ, UK; 2 Digital Catapult, London NW1 2RA, UK; alex.gluhak@digicatapult.org.uk; 3 Department of Computing, Imperial College London, London SW7 2AZ, UK; j.mccann@imperial.ac.uk; 4 School of Engineering, Cardiff University, Cardiff CF243AA, UK; BeachTH@cardiff.ac.uk

**Keywords:** water usage disaggregation, water monitoring, disaggregation algorithms, machine learning, water management

## Abstract

Water monitoring in households is important to ensure the sustainability of fresh water reserves on our planet. It provides stakeholders with the statistics required to formulate optimal strategies in residential water management. However, this should not be prohibitive and appliance-level water monitoring cannot practically be achieved by deploying sensors on every faucet or water-consuming device of interest due to the higher hardware costs and complexity, not to mention the risk of accidental leakages that can derive from the extra plumbing needed. Machine learning and data mining techniques are promising techniques to analyse monitored data to obtain non-intrusive water usage disaggregation. This is because they can discern water usage from the aggregated data acquired from a single point of observation. This paper provides an overview of water usage disaggregation systems and related techniques adopted for water event classification. The state-of-the art of algorithms and testbeds used for fixture recognition are reviewed and a discussion on the prominent challenges and future research are also included.

## 1. Introduction

The global use of water is increasing at a rate faster than can be satisfied with current usable water supplies [1]. While irrigation and electricity generation dominates water usage in developed countries [2], household water conservation still represents an important factor in ensuring sustainability of fresh water reserves on our planet. In the US, for example, nearly 10% of fresh water consumption can be attributed to domestic use [3]. In the UK, each person uses about 142 L of water each day with the average household using 349 L of water per/day [4]. Even though domestic water usage only accounts for 10%, achieving efficiencies and understanding this usage is of particular importance to water utilities. Key reasons for this include [5]; (a) differing the requirement of construction of expensive capital assets, such as reservoirs or boreholes; (b) problems relating to efficiently supplying sufficient water quantities in often increasing dense urban area that are at considerable distance from existing water resources; and (c) achieving a pressure reduction in a given urban region and thus extending the lifetime of assets. In the UK, urban water systems make up 6% of CO_2_ emissions, with 89% coming from heating water and the remaining 11% coming from treating and pumping water [6]. These figures do not take secondary water usage into consideration; this is usage that would be attributed to the food or energy production that we as individuals consume in our daily lives. That is, food production is said to consume 3496 L of water per day per person and, within the home, further food preparation consumes 10% of water usage—remarkably, 50% of this is then wasted [7].

Due to humanity’s dependency on water, its shortage can cause economic downturn and ultimately civil unrest; it is of no surprise that a considerable amount of research has been conducted to understand water use and, in particular, water use in a residential setting. Previously, a multitude of approaches have been utilised, in order to gather and interpret household water usage data.

Understanding water use in residential environments provides considerable value to water utilities, policy makers, and water users alike. From a water utility perspective, detailed knowledge of household consumption patterns can enable the predictive management of water supplies, leading to significant savings in the distribution and storage of water in networks [8]. It also provides the foundation for new water demand management strategies aiming at increased water conservation or shifting water usage peaks, and it can even contribute to improving the detection of costly leaks in water distribution systems. Policy makers can utilise such information for better conservation planning and to devise more adequate response strategies in periods of water shortage [9]. From a residential water user perspective, information about their own water usage behaviours can lead to identifying wasteful water usage habits or water leaks at premises, providing the foundation for a sustainable behaviour change and cost savings on water bills [10].

In the following sections, we briefly review existing approaches for contextualising water use in residential settings. Our focus is on techniques for assessing water usage in indoor settings, as this field of research has reached a critical mass of works that motivates an extended review. We classify them according to their purpose, underlying technology assumptions, and working principles, and discuss advantages and disadvantages of these methods. The paper then ends with an identification of further opportunities to overcome these limitations and identifying further research directions for work in the field.

## 2. Understanding Water Usage

For a domestic setting, there are a variety of questions that can be asked, which can lead to insights about the water use of a household at different levels of granularity:

- How much water is being used?
- What water is being used—is it hot or cold water, fresh water or grey water?
- When is water being used—how does the water use change over the day, week and seasons?
- Why is water being used—what are the activities related to water use?
- Where is water being used—at which fixture or fixture type is water being abstracted from? Are these indoor or outdoor uses?
- Who is using the water—in a multi-occupancy household or building, how can water use be attributable to individuals?

When unravelling insights about domestic water use, it is obvious that not all questions may be relevant for all stakeholders and that different exploitations may require a specific water usage contextualisation to be obtained.

From a water utility perspective, there are different motivations as to why obtaining insights into household level information is important. At the most basic level, water usage information on individual households is important for billing purposes. Aggregate monthly or even yearly water usage information may be sufficient for water utilities to obtain adequate compensation for their domestic water supply. Moreover, in order to achieve water distribution network efficiencies and to understand investment levels, the relative costs and benefits of consumption are required; these can only be estimated by determining households’ water use [10]. This, in turn, impacts on water pricing mechanisms. This information is useful to manage resource constraints and future demands. From end-use analysis, utilities can gain insights into how new generation appliances will positively affect demand, and can thereby avoid oversupply before it is needed. Having information of water usage at higher time resolutions from households in a district metered area (DMA) can be helpful in identifying water leaks in the distribution network [11], leading to significant savings for the water utility, such as the water wastage itself and the associated energy required to treat and pump it around the distribution system. Detailed knowledge of water use across different times of the day, weekday and weekend demands, as well as seasonal variations facilitates water forecast demand modelling, which can used to better schedule water availability across the water network, thus saving energy costs for pumping, and ensuring appropriate supply levels [10].

From a policy maker perspective, a more detailed understanding of residential water use is required to support the identification of water saving opportunities and to devise effective strategies and validate their effectiveness. For this, a breakdown of the water use in the home, based on usage activities or water fixture types is required, as well as an understanding of the temporal change of domestic water usage behaviour [6]. Quantifying water saving opportunities naturally also applies to hot water use [12], where additional energy is wasted in heating up the water. A policy maker also draws upon residential water usage information in order to regulate water prices and to influence how water use is communicated to end users by the water utilities [13].

Domestic water end users are primarily interested in cutting their water bill or to contribute to personal sustainability targets [14]. Therefore, they require more detailed knowledge on water usage activities to understand what water savings opportunities exist and how they can adapt their water usage habits. End-use studies [6] provide simple guidance on how water use can, on average, be broken down into usage categories and activities at home. These insights can be combined with actionable advice given by water companies and environmental organizations. While such end-use studies provide a good view on a national average, there are considerable variations in water usage behaviour across households in different regions, even within similar communities [15]. More personalised information is thus required in order to make water demand management more effective within individual households.

Feedback systems have recently emerged as a more promising alternative to support sustainable behaviour change in domestic settings. While the initial bodies of work focused primarily on energy, in addition, water related eco-feedback systems are beginning to emerge [16,17]. These works indicate increased water saving potential where end users are provided with more personalised and timely feedback about their water consumption. Froehlich *et al.* suggest that a detailed breakdown of water use to fixture types or even the attribution of water use to individual persons in a household could be effective [16]. Kuznetsov *et al.* propose the use of “live” *in situ* feedback during water use at the fixture to influence a user’s consumption behaviour [17]. An understanding of detailed water usage in a home can also highlight existing water wastage due to leaks in the domestic water infrastructure and inefficiency of fixtures, leading to longer term cost savings for the resident if appropriate actions are taken. While the above discussion is non-exhaustive, it highlights the benefits and potentials of obtaining more detailed insight into domestic water use of different stakeholders, and shows the spectrum of types and granularity of information that are required to obtain these.

However, it is worth mentioning that the increasing adoption of eco-friendly appliances is fortunately driving towards theoretical lows for indoor water use. Even if users can be educated to use water wisely, it is hard to change personal habits in a way that can yield a significant impact on water resources. Regarding outdoor residential usages, some efforts have been made at the regulatory level to improve landscape water management. For instance, a Model Water Efficient Landscape Ordinance [18], has been developed and updated by the Department of Water Resources in California. Industry and research should refocus on the conservation of outdoor water use, which has not received as much attention. Across different study sites, more than 50% [19] of residential water use is outdoor, and outdoor use reaches the peak in the hot season, when pools and gardens require water [20]. This suggests that, in terms of disaggregation, outdoor usages involve large volumes of water but are easier to identify because it is limited to the two usages mentioned above. For these reasons, this paper will focus on indoor settings, which have much larger variability and the disaggregation of activities is, therefore, more challenging.

The rest of the paper is structured as follows: Section 3 introduces the approaches to contextualize domestic water usage analysing the current panorama of metering, introducing the concept of disaggregation and then a short taxonomy of the sensing possibilities adopted so far in scientific literature. The Section 4 is focused on the statistical learning and classification of water events and reports a number of existing works in the field. Finally, Section 5 contains a discussion of issues and challenges that still narrow the applicability of water disaggregation in real world scenario.

## 3. Approaches for Contextualizing Domestic Water Use

In the following we examine the state-of-the-art approaches that are used to contextualise domestic water use.

### 3.1. Assessing Individual Water Consumption

An effective way of measuring household level water consumption is through metering the water supply at the premises of a customer. Only about half of UK households currently have a water meter installed [21]. The vast majority of these meters are not Internet-connected, and require a manual readout by the water company or the customer. Meter readouts often take place on an annual or monthly basis, in order to estimate the domestic water bill. Non-metered customers are charged an amount that is proportionate to the rateable value of the property [22]. This results in an annual flat rate that does not take into consideration the size of the household [23]. Automated meter reading (AMR) or smart meter reading provides the ability to automatically capture water usage information at more regular intervals. In their most basic form, such meters do not require a connectivity infrastructure. They act as standalone meters that can be read through some wireless channel in a walk-by (e.g., handheld devices) or drive-by fashion (e.g., utility service vehicle). A more effective way is connecting AMR/smart meter devices via a dedicated metering infrastructure to the utility company, or via existing communication networks available at the household (e.g., phone line, Internet router). While this comes at increased costs and complexity, it removes the burden of relying on physical proximity for retrieving the meter readout, theoretically allowing near real-time reporting of metering information. In practice, meters are typically monitored on a daily, hourly basis or 15 min basis [24], as dictated by the costs for data communication and data storage, respectively.

In contrast to AMR devices, which provide only simple reporting functionality, smart meters can provide bi-directional communications. Depending on their extended capabilities, smart meters can provide some configuration options to the utility company, such as the configuration of the reading interval or other system settings. Some smart meters can be even interfaced to in-home displays or smart home platforms, providing residents with information on their current or historic water use [25].

Despite their advanced metering capabilities, current smart meters are only capable of answering how much water is being used and when. Breaking down the residential water use to more fine-grained levels, e.g., fixture level use, requires higher resolution readings combined with external data analytics and possible additional instrumentation. Recent work in the field of energy metering calls for an evolution of smart meters to become “cognitive meters” [26] that are able to disaggregate the water use within the metering device. While basic features, such as household leak detection on smart meters, is already feasible [27], this vision still requires some further advances in the field.

Interestingly, the scientific community has been working since the 1990s on approaches for water use disaggregation at the household level. In the following, we will examine these works and identify strengths and weaknesses of these and current gaps.

### 3.2. Non-Intrusive Disaggregation of Domestic Water Use

Disaggregation of water use represents an active field of research as it provides important insights for the contextualisation of domestic water use, which simple metering cannot answer.

On a high level, disaggregation allows domestic water use to be broken down into fixture categories, which identify the amount of water consumption of individual fixture types in a household. Typical fixture categories for indoor use are shower, bathtub, toilet, and faucet, as well appliances, such as washing machines and dishwashers. Typical outdoor fixture categories are exterior hose bib, swimming pools and irrigation systems. Most current end use studies provide insights on residential water use at the level of the fixture category. Sometimes, however, it is necessary to distinguish between hot and cold water use, and the location (room, indoor or outdoor) where the water is being used. Such water usage break down requires knowledge of water use at individual fixtures or even valves (in the case of hot and cold water).

A further level of contextualisation is the attribution of water use to individual residents in a multi-party home or the mapping of water use to individual activities (e.g., washing hands, cleaning teeth, watering the garden, *etc*.). The latter contextualisations are particularly hard to obtain.

One of the most common methods for deriving a breakdown of domestic water end use information is through manual data collection acquired by consumer surveys, diaries/self-reports and *in situ* observations in domestic living environments. Such studies are able to capture a diversity of information, even detailed information that is sometimes very difficult to capture, such as water usage activities. However they tend to be very labour intensive and do not scale well for longitudinal analysis (over longer periods of time for larger populations). Online survey tools, such as the Water Energy Calculator [28], have made it easier to reach wider audiences [6], however, such studies rely on the truthfulness of the persons participating in the studies. Despite their best attempts at being honest, users often reflect perceptual bias or may accidentally misreport relevant information [29]. Furthermore, self-reports and surveys are not able to capture the exact amount of water use and represent only estimates that have to be complemented by more detailed metered water use. Researchers in the field have therefore looked into instrumenting households and applying data analytics solutions to measured data traces in order to gain a better insight into water usage patterns.

A very accurate but inefficient way to obtain such insights is through extensive instrumentation of a household. Each individual fixture or even valve can be instrumented with a flow meter. Such deployments are mainly limited to testbed settings [30,31,32], in order to establish a ground truth for other experiments with less intrusive techniques. It is not difficult to see that such an approach is, not only cumbersome and costly in terms of deployment, but highly intrusive. Such a case can be seen as analogous to intrusive load monitoring in the energy domain [33].

In order to overcome the above limitations, a variety of non-intrusive monitoring approaches have been proposed, which are able to perform water disaggregation based on data obtained from a single sensing point or from a limited set of sensing points deployed at strategic locations of a residential water pipe infrastructure and/or rooms of a residences.

Non-intrusive water use disaggregation approaches differ in terms of the underlying sensing process and the classification techniques that they utilise to discern water usage patterns from the sensed signals.

In the following, we first discuss commonly used sensing processes, and highlight key features and differences of these. Based on the knowledge of these processes, we subsequently structure our discussion on the classification approaches and cover the main challenges, strengths, and weaknesses of these.

### 3.3. Sensing

The sensing process for non-intrusive water use disaggregation approaches is determined by a variety of factors. A first key discriminator is whether a single or multiple sensing points are required for a residential setting.

While single-point sensing solutions are typically based on a single modality, multi-point approaches can utilise one or more different sensing modalities. Approaches that utilise a single sensing modality are referred to as mono-modal sensing approaches, while approaches that utilise multiple sensing modalities for water use disaggregation are referred to as multi-modal.

Depending on the nature of sensing modality the sampling frequency may greatly vary. Low frequency approaches typically operate in sub-Hz regions, while high frequency approaches can require up to several kHz sampling of the sensing signal. The sampling frequency determines the data rate, which has an impact on processing, storage, and communication requirements.

The sensing process also typically determines whether associated water volumes of water usage events can be determined.

**Figure 1:**
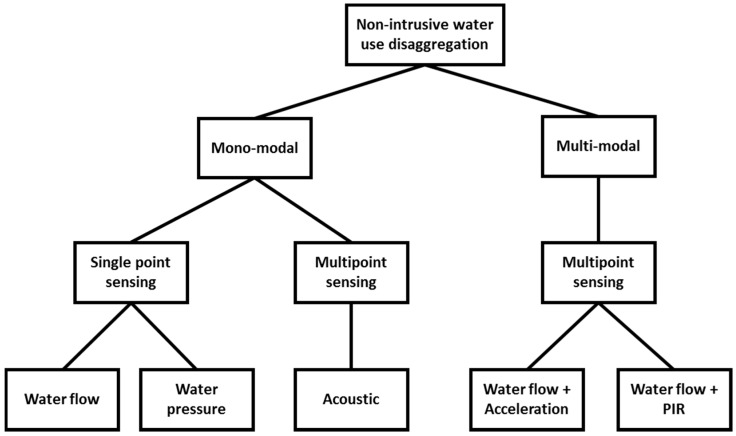
Overview of the different sensing modalities for water disaggregation (PIR stands for Passive Infra-Red).

Figure 1 shows an overview of the different sensing modalities utilised in current approaches that can be found in literature.

Flow meters are the most commonly-used sensing modality [34,35,36], as they can be fitted outside of the premises of a customer and are, thus, the least intrusive instrumentation option from an occupier’s perspective. They directly measure the volume of utilised water usage activities, which forms the basis of customer billing. Approaches based on flow meters require relatively low sampling rates, e.g., one sample every 5 s.

If instrumentation inside of a customer premises is acceptable by both: By users (in terms of privacy, wiring and radio emissions), and by water suppliers (in terms of cost/benefits), and more sensing options do exist. One of the most promising alternatives for single point sensing is water pressure [31,37,38]. A pressure sensor is typically attached to the residential water pipe infrastructure (e.g., hose bib, bigot, *etc*.) and is able to measure changes to pressure caused by water usage at different fixtures. Compared to flow meters, approaches based on pressure sensing require higher frequency sampling (500–1000 Hz) in order to reliably detect signatures of water usage events. While they cannot directly measure the volume of water usage activities, the volume can be estimated with additional techniques.

Both water flow and water pressure are modalities obtained by inline measuring of the water piping infrastructure. As they are directly in contact with water, such sensors require a thorough approval process to be authorised for deployment by water authorities [39]. Furthermore, the installation of such sensors proves to be more complicated.

Acoustic based sensing [30] does not require the insertion of sensors into the piping infrastructure. Instead, acoustic sensors are simply placed on top of the pipes at a few strategic locations. In contrast to flow and pressure based sensing, which require only a single sensing point, multiple acoustic sensing points are required to be deployed for reliable disaggregation. Acoustic event detection algorithms are able to determine water usage events; however, they are unable to infer the volume of the utilised water data. Like pressure based sensing approaches, acoustic sensing approaches require higher frequency sampling (around 4 kHz).

Finally, several multi-modal sensing approaches have emerged that aim to improve the disaggregation accuracy of flow meter based sensing. Kim *et al.*, in their non-intrusive autonomous water monitoring system (NAWMS) [40], utilises a flow meter at the main supply and accelerometers deployed at individual pipes leading to fixtures to disaggregate water use to individual fixtures and performs flow rate estimation for each fixture. It uses lower sensing frequencies for the flow meter, but requires a 100-Hz sample rate for the accelerometer signal. WaterSense [41] utilises passive infrared-based motion sensors deployed in rooms with fixtures to improve fixture level classification of flow meter based disaggregation. It also can determine the volume of water used during each classified usage event. WaterSense requires a sample every 2 s for flow and one every seven seconds for the motion sensor. The latter is only processed when motion is detected.

## 4. Classification of Water Events

As previously discussed, the approaches for water use disaggregation make use of a variety of different sensing modalities. The sensed signals are then subject to pattern analysis by applying classification techniques in order to identify the corresponding water usage events. Depending on the nature of the sensing modalities, different classifiers are utilised. These classifiers can be further divided into discriminative and generative ones. In the following, we structure our discussion on the classification techniques according to the utilised sensing modality.

### 4.1. Water Flow Based Methods

A key assumption of non-intrusive monitoring approaches based on flow meters is that the use of a particular water fixture or fixture type causes a distinct flow pattern in the residential pipe infrastructure, which can be observed by a single sensor point. By applying pattern recognition algorithms to the recorded time series data, water usage events for the specific water fixture or fixture type can be identified.

**Figure 2:**
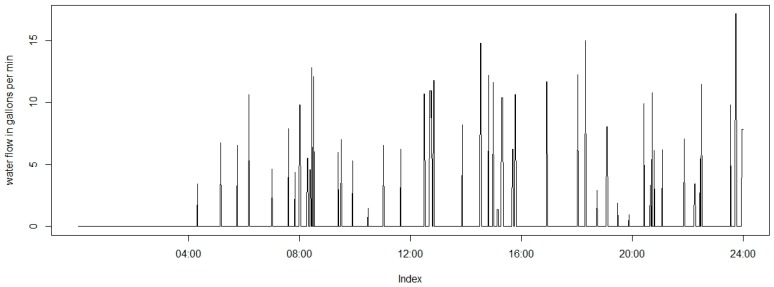
One-minute averaged flow over a full day.

Water flow in households is typically modelled with Poisson rectangular pulse as described in [42]. Figure 2 shows a 24-h dataset generated from the aforementioned model. The number of events in a unit of time follows a Poisson distribution, while duration and intensity have their own mean and variance. All the parameters should be adjusted by time of the day with diurnal multipliers. The observed flows are directly related to the volumes. Reported figures about water volumes and flows are shown in Table 1 [34,43].

Non-intrusive monitoring techniques based on flow meters face the following difficulties:

- Irregularities of flow patterns for some fixture types. Some mechanically driven water valves, such as in a washing machine or dishwasher, usually exhibit more regular water usage patterns, unlike faucets or showers where human users have the ability to vary the amount of flow and duration significantly. Even the more regular flow patterns of washers can show variation based on a diversity of different water saving programs and washing cycles that are available in modern-day devices.
- Similarity of flow patterns among instances of the same fixture type. Many homes have multiple toilets and/or faucets, which may be located in different rooms. This makes it challenging to identify an individual fixture if multiple instances of the same type exist [34,38].
- Overlapping of flow patterns. Often, fixture use can occur concurrently, resulting in overlapping patterns in the observed water flow that are difficult to disassemble [34,36]. A typical example is a person washing their hands after a toilet flush, while the water tank refills. In multi-occupancy homes, such events are even more frequent. This superposition of patterns causes additional challenges for pattern recognition techniques, and this becomes more severe with an increasing number of overlapping water usage events. Some of these challenges can be overcome by adopting different sensing modalities.

In terms of volumes consumed inside homes, Table 2 reports indoor household use by fixture based on a large study involving more than 23,000 homes in North America [44]. The statistics and figures in the report are affected by regional factors, and cannot be considered representative of all Western countries, or of all the United States, but the breakdown of indoor usage, expressed in percentage, is not expected to differ substantially.

#### 4.1.1. Discriminative Classifiers

Flow trace analysis is one of the first automated techniques to infer water usage from single flow meter readings. It was initially proposed by Dziegielewski *et al.* [45], and is currently the most widely-used technique for identifying water usage events in the water industry due to its maturity and the availability of commercial service offerings based on it. Flow trace analysis relies on the fact that domestic water use exhibits common patterns that are distinctive enough to discriminate water usage events of different fixture types. Through analysis of aggregate data flows captured by a single flow meter by visual comparison with a database of water event signatures, or by the use of simple decision-tree based classifiers, the current water source for these water usage events can be determined.

A first extensive study that utilised flow trace analysis was presented by DeOreo *et al.* [34]. The authors performed a collection of signature flow traces for each fixture inside of 16 homes at a rate of one sample every 10 s using a flow meter. The signatures encompassed nine distinct example categories and where stored into a database as reference signatures for later analysis. Then the 16 houses were monitored over a period of three weeks each. Using the signatures, data-flow traces were determined based on visual analysis. When a type of flow was identified, it was isolated in a window and the integral of the flow rate over this window provided the volume of water used for the event. Overall, 10,000 water usage events were identified.

In order to simplify the analysis, a signal-processing algorithm was devised that utilised different feature sets derived from the flow meter measurements. The algorithm parameters were derived from the labelled empirical database and included features, such as peak flow, duration, volume, flow rate change over time and time of the day cues. The authors however did not provide any assessment of the performance of their solution.

The two market leading commercial tools, TraceWizard [46] and Identiflow [47], are also based on the principle of flow trace analysis. According to a previous review by Nguyen *et al.* [48] for these two systems, both use decision tree based classifiers and require a time-consuming and labour-intensive process to perform offline fixture disaggregation.

TraceWizard is reported to apply an algorithm that interprets data based on simple boundary conditions. Examples of these boundary conditions include start time, stop time, duration, volume, peak flow rate, the most common flow rate, and how often this most common flow rate occurs during the duration of the event. However, the performance drops very quickly to 24% when two water fixtures are used at the same time or 0% when three or more were used. Similarly, Identiflow has the same deficiencies. It uses a decision tree algorithm to deconstruct a flow trace data series into water end use events and achieves an accuracy of 74.8% in terms of the correctly-classified volume. As it relies on fixed physical features of various water-using devices, such as volume and flow rate for disaggregation, the final classification accuracy is greatly dependent on the existing types of water devices.

In recent work, Dong *et al.* [49] propose a Deep Sparse Coding based Recursive Disaggregation Model (DSCRDM), which is particularly suited for low sample rate water consumption disaggregation. Their algorithm is inspired by work in the energy disaggregation domain on Discriminative Disaggregation Sparse Coding [50], which the authors extend using a recursive decomposition structure to perform the disaggregation task. Starting from the total measured consumption, the consumption of each fixture type is disaggregated in a step-wise approach during which a current device is distinguished from other residual devices through a discriminative dictionary. It utilizes only one sample every 15 min. It achieves an average accuracy of about 52% and a normalized Disaggregation Error of 74% for classifying fixture type usage events, such as faucet, dishwasher, toilet, humidifier, cooler, hot tub, shower, bath, irrigation, and swimming pool. While the accuracy is lower than comparable state of the art flow trace solutions, the approach works at much lower data rates. Piga *et al.* propose a novel algorithm [51] based on sparse optimization, which the authors claim can be used to disaggregate both water and energy consumption data. The approach is based on the assumption that the power/water consumption profiles of each appliance are piece-wise constant over the time and it exploits the information regarding time-of-day probabilities whereby a specific appliance/fixture is likely to be used. The algorithm treats the disaggregation problem as a least-square error minimization problem, with an additional (convex) penalty term aiming at enforcing the disaggregate signals to be piece-wise constant over the time. The proposed algorithm is able to reconstruct the consumption trajectories over time and has shown excellent disaggregation performance using an energy data set with four overlapping household appliances. While the algorithm is likely to perform well on water use patterns of household appliances, it is unclear how well it will do on more irregular water usage patterns, such as tap use or showers.

#### 4.1.2. Generative Classifiers

The work of Fontdecaba *et al.* [36] assumes flow meter data at a rate of a reading every 5 s. It considers a common generative model for all households, which models water usage classes (toilets, washing machine, kitchen sinks, bathroom sink, dish washer, shower) as probabilistic models with multivariate Gaussian distribution. A maximum likelihood estimator is utilised to select the right water usage class based on 10 indicators derived from the flow meter data. The algorithm achieved an overall classification accuracy of 70% for water usage classes and 68% for water volume, considering sample data obtained from eight households over a period of three months. The algorithm was assed only for non-overlapping usage events and had difficulties in accurately classifying water using appliances, such washing machines and dishwashers.

In Reference [48], Nguyen *et al.* investigated the use of a Hidden Markov Model (HMM) based classifier for water end-use event classification. They found that HMM alone did not provide sufficient classification accuracy and added extensive context information to fine-tune the classifiers, based on time of day, likelihood of occurrences of events, and assumptions of event durations and flow boundaries and volumes per event. The resulting approach was a hybrid analytical method employing an HMM with over 100 states with a Dynamic Time Warping algorithm and event probability techniques, resulting in a multi-layer classifier. The classifier was able to disaggregate water usage events for tap, dishwasher, washing machine, shower, bathtub, toilet and irrigation. The classification for most events was nearly 90% for non-overlapping events, apart from irrigation and bathtub, which the algorithm had difficulties recognising accurately. A bathtub was often confused with a long shower; likewise, irrigation difficulties were due to irregular patterns.

The same team performed analysis of overlapping water events. In order to deal with concurrent events, they proposed a new filtering method [52], which smoothens a combined event to any desired level based on examination of gradient change along the sample, in order to make different dissection decisions. The filtering determines a base samples and subsamples. Both base event and subsamples are classified by an HMM, based on their likelihood. Subsamples require an additional threshold or are broken into further subsamples using the same filtering technique. The base event is classified based on likelihood without a threshold. The evaluation looked only at the fairly small number of 20 combined events (between two and three concurrent occurring events) and was able to perform with a classification accuracy of 88%. While the results look promising, the sample size was too small for it to be considered significant.

### 4.2. Water Pressure Based Methods

Water pressure based disaggregation approaches exploit the fact that a building’s piping infrastructure forms a closed loop pressure system with water held at a stable pressure throughout the infrastructure when no water is flowing. In the case where no pressure regulator exists at a household’s mains supply, this pressure may be subject to minor variations depending on the water neighbourhood water demand.

The opening and closing of valves along this pressurized piping infrastructure leads to the generation of pressure waves, which result from the rapid change of velocity of the water in the pipes. The magnitude of the surge in pressure waves is independent of, and much greater than, the operating pressure and the resulting transients can have a positive or negative rate of change depending on whether a valve is being opened or closed. The signatures of these transients depend on the valve type and its location in the home pipe network, providing excellent discrimination capabilities to even distinguish among two fixtures of the exact same model. To a lesser extent, the signature is also influenced by the way the valve is opened. The magnitude of the pressure drop and resulting shockwave is dependent on the relative location of the sensor’s deployment point to the source of the event and the speed that the valve is opened or closed, but the shape of the signature does not change.

Non-intrusive monitoring techniques based on flow meters face the following challenges:

- Overlapping of flow valve events: The magnitude and shape of the transients are altered by the overlapping water use events. This has an impact on the ability to perform accurate event segmentation, especially for events that occur very close together.
- Generalisation: Due to the dependency of the sensing system on the piping infrastructure topology, sensor placement and fixture types, a calibrations phase for each valve during installation may be needed. This makes the deployment of the solution more difficult without auto-calibration methods.
- Accuracy of flow estimation: The amount of water flow cannot be directly determined, and requires and estimation to be performed based on changes to the pressure. For this to work, additional calibration steps are required to approximate the behaviour of the piping infrastructure.

HydroSense [38] is the first approach to propose non-intrusive water use disaggregation based on pressure sensors. HydroSense requires a pressure sensor to be installed on an available water hose bib, utility sink faucet, or water heater drain valve. In their work, Froehlich *et al.* collected samples of valve open and close events of all fixtures in the home at a 1 kHz sampling frequency with a pressure sensor from 10 test homes, in order to extract signatures for these water usage events. Based on the collected data set, they developed an approach that allows the classification of fixture open and close events in a three-step approach: (1) valve event segmentation is based on a FIR low pass filter over a 1-s time window and determined based on a threshold over the derivate of the filtered signal; (2) valve event segmentation determines valve open and valve close events using a hierarchical classifier; (3) fixture classification, which maps valve open/close events to an individual fixture with a template based hierarchical classifier with different distance metrics. Flow estimation is based on an equation that can approximate the flow with a change in pressure by measuring the difference between the pressures at the onset of a detected valve open event to the stabilized pressure at the end of the segmented valve open pressure wave impulse. In their experimental setup, they achieved 97.9% aggregate accuracy for identification of individual fixtures, and flow rate estimation errors between 5% and 22%. Their work only performed offline classification of isolated fixture usage events and considered valves that were fully opened/closed.

In later work [37], HydroSense is further extended by Larson *et al.* to be able to, not only to perform valve fixture level classification, but also determine the valve at a fixture responsible for the water use, e.g., discriminate between a hot and cold water tap. Their work assessed two different classifiers during the third step of HydroSense, which maps identified valve events to individual fixtures/valves: (1) a template based classifier from their earlier work and (2) an HMM based classifier. A two-state HMM with four diagonal covariance Gaussian mixtures per state was found to provide the highest accuracy due to the sparse training set available. The study showed no notable different between the two classifiers with fixture level (template based 96.4%, HMM 95.2%) and valve level (template: 94.1%, HMM: 92.3%) with the template classifier having a slight edge. The authors, however, suspect that the HMM, being a more stochastic classifier, may be more robust in dealing with partially-turned fixtures under realistic conditions.

The authors use a sparse data set of fixture trials collected under idealistic conditions at 10 households (775 fixture events collected from 76 valves/51 fixtures) for training and assessing their classifiers. This made it difficult to draw conclusions on how HydroSense operates under realistic conditions. A study [31] with a more robust data set was carried out with HydroSense where the authors collected 15,000 annotated ground truth events from five homes in period of five weeks; however, to achieve this result, the authors needed to build a data collection toolkit, which took three months before being deployed. This toolkit comprises visible and invasive sensors, together with off-the-shelf meters, which have been hacked to fit the experiment requirements.

Their algorithm used ground truth labels for event segmentation, and focused on a probabilistic approach for valve event classification using Bayesian estimation, which is an approach that is inspired by the dynamic Bayesian models used in speech recognition (where instead of recognizing words, valve events are recognised). It consists of the following parts: (1) template matching using similarity matching algorithms; (2) a language model to determine likelihood of a sequence of valve open/close events to identify event pairs; (3) extract features from paired tuples and compare them to a probability distribution; and (4) combine probabilities to select the most likely sequence. Using a single pressure sensor per home, their algorithm was able to disaggregate valve, fixture, and fixture type at 70%, 90% and 96% percent accuracy with a single sensor, which rose to 82%, 93% and 97% if a second sensor was deployed on the hot water piping infrastructure. The algorithm also showed acceptable performance in the presence of two overlapping usage events. To be practically deployed and usable, the algorithm requires some staged training data for each fixture and automatic segmentation of events. While previous work in HydroSense showed that automatic segmentation was possible with non-overlapping events, classification accuracy is likely to be worse due to segmentation errors in overlapping situations.

### 4.3. Acoustic Based Methods

**Figure 3:**
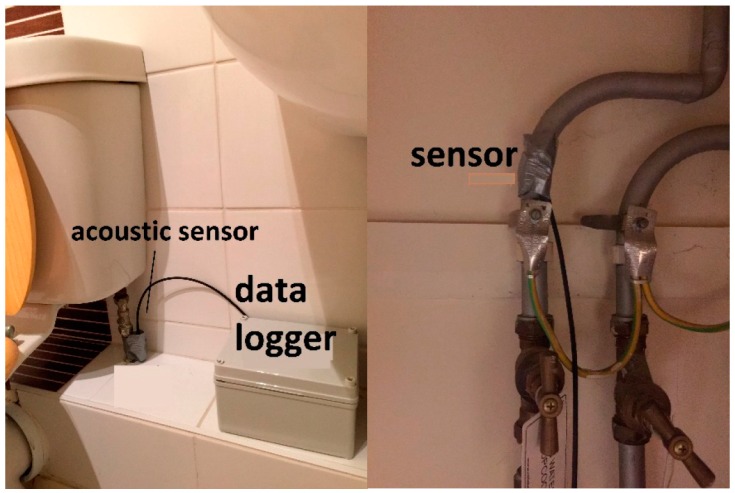
Acoustic sensors deployment for in-house water monitoring.

Acoustic event detection provides another alternative for disaggregating water use in a residential environment (Figure 3). The assumption of such methods is that water usage events can be derived from audio signals captured by microphones that are placed at strategic locations along the water piping infrastructure of a home.

An approach based on acoustic sensing is presented by Fogarty *et al.* [30]. Four acoustic sensing units are placed in the basement of the home, one on the cold water pipe, one on the hot water pipe from the heater, and two sensors on the waste water pipes. The sensing units were Mote class devices equipped with microphones and performed intermittent high frequency audio sampling (1000 samples in 0.25 s, every 2 s). From these 1000 sample windows, features are extracted in the form of zero-crossing rate and the root mean square. These features are fed into a hand-crafted hierarchical classifier that exploits knowledge of activity patterns and interdependencies across the two supply and two drainage pipes for classifying water usage activities into the categories of washing machine, dishwasher, shower, toilet, kitchen sink, and bathroom sink use. While not able to determine the water volume or duration of the water usage events, the proposed classifier was able to determine the usage of a particular fixture with an accuracy between 70%–100%, depending in the fixture type. As only performance data for isolated usage activities were presented, it is unclear how the classifier performs for overlapping usage events. The proposed approach faces further two limitations: Short usage activities below 10 s, such as shot sink events, cannot be accurately determined; and the approach is prone to error introduced by systematic noise sources in the home, such air-conditioning units. The authors suggest placing the sensors far away from the noise sources to mitigate these issues. This initial work demonstrates the viability of acoustic based sensing for water disaggregation; however, the limited assessment leaves questions as to how the approach can scale to different environments.

### 4.4. Multi-Modal Methods

**Figure 4:**
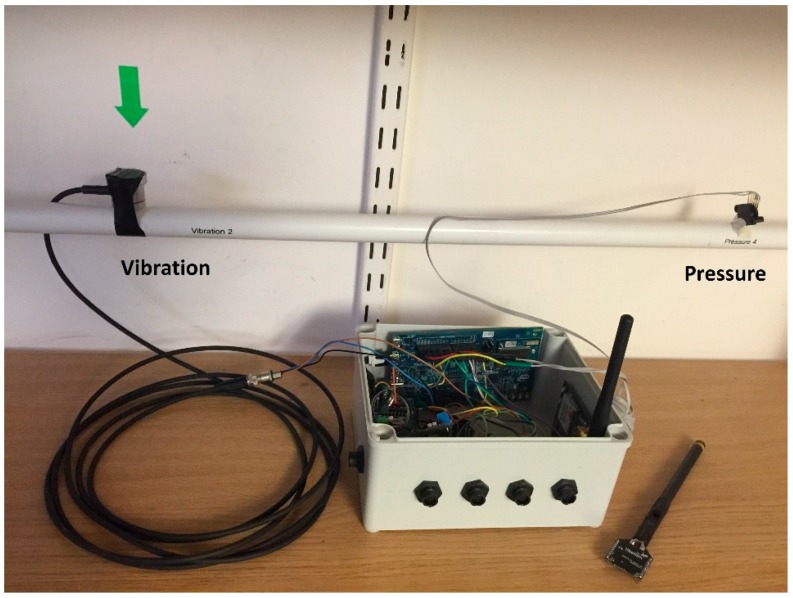
Multi-modal (pressure on the right and vibration on the left) sensing deployment on a pipe. Sensors are wired to a gateway for data collection, analytics and transmission.

Multi-modal methods exploit multiple sensing modalities in order to perform water usage disaggregation. They are able to exploit a richer set of features derived from independent sensing streams to perform classification of water end usage events. Figure 4 shows a lab setting with multiple sensors (pressure and vibration) deployed on a pipe and wired to a gateway.

One of the first multi-modal methods was NAWMS [40], which was proposed by Kim *et al.* It exploits the fact that water flow along the piping infrastructure leading to a fixture causes vibrations of the pipe that can be measured by an accelerometer attached to the piping infrastructure. NAWMS utilises a flow meter at the main supply of the home to measure the total volume, and performs disaggregation based on signals derived from accelerometers placed on the pipes leading to individual fixtures. It assumes that an accelerometer per fixture is needed. More specifically, NAWMS samples at a frequency of 100 Hz, and considers the variance of an accelerometer over 50 samples as a feature. It proposes an adaptive auto-calibration procedure, which attempts to solve a two-phase linear programming and mixed linear geometric programming problem for estimating parameters necessary to translate the detected vibrations to actual water flow. It achieves an accuracy of about 90% for volume disaggregation, and claims to be independent of the fixture attached to the pipe. NAWMS has only been tested on a three-pipe lab testbed. Deployment in a real environment appears to be more challenging, as it requires one accelerometer per pipe. The optimisation problem to solve using an algorithm needs to be configured to a specific pipe structure, as the fitting model requires tuning to the pipe material and diameter.

WaterSense [41] represents a more recent work by Srinivasan *et al*. Their main idea was to utilise knowledge about human presence near water fixtures as additional insight to perform fixture level classification. It exploits that fact that fixtures with similar flow signatures are in different rooms and that fixtures in same room have different flow signatures. WaterSense requires PIR sensors in each room where water fixtures are installed, and a flow meter at the main water supply of the home. It samples the flow meter at a frequency of 2 Hz and draws presence events from PIR once every 7 s if a presence is detected. It employs a 3 tier unsupervised inference algorithm, where tier 1 detects water flow events based on edge detection of the flow meter signal, tier 2 performs clustering based on rooms using a Bayes Network clustering approach, and tier 3 performs fixture determination based on event duration and frequency. Fixture disaggregation is limited to sinks and toilets. It shows an accuracy of 86% for fixture event classification, and 80%–90% accuracy for individual fixtures. Unlike all previous approaches, WaterSense is unsupervised and does not require any training data. However, it has difficulties in classifying overlapping usage events if the same fixture type is used in different rooms simultaneously. It also cannot discriminate between multiple fixtures of same type in the same room. Unlike all of the previously-discussed work, Ranjian *et al.* were the first to consider disaggregating water use, not on the fixture level, but to attribute water use to individual users in a household [32]. In their research, the authors explored whether room-level tracking of individuals is accurate enough for user attribution of fixture use of both for water fixtures and energy. They instrumented a test home with underfloor Radio Frequency Identification (RFID) readers embedded in each doorway for house- and room-level tracking, and 15 RFID readers at individual fixtures for high accuracy, which were able to track individuals that wore an RFID ankle bracelet. Fixture level use was directly inferred by flow meters attached to each individual fixture. The home examined different house, room, and fixture level tracking, and assessed the use of simple heuristics based on people history of fixture usage to resolve ambiguous situations. Performance of house-level tracking could be improved to 60% with heuristics, room-level up to 87%, and coordinate-level up to 97%. The work showed that room-level tracking of users in homes can provide a good accuracy for user attribution of water usage activities in the home. However, the approach using RFID tracking with ankle tags and underfloor readers is very intrusive for everyday deployment contexts.

### 4.5. Summary of Approaches

To give a picture of the pros and cons for the different approaches, and starting from a subset of selected works listed in the previous sections, Table 3 reports a summary in terms of output/performances, resilience and installation.

## 5. Discussion on Issues and Future Challenges

Water usage disaggregation is the equivalent of non-intrusive electricity load monitoring, applied in the water domain, but with an important difference: While electricity outlets can be monitored with non-invasive, out-of-the-box meters, water fixtures are, in general, unpowered and more difficult to wire to a data communication infrastructure. This entails battery-operated instrumentation and, in turn, constrained communication capabilities. Moreover, when dealing with supervised classifiers, a necessary step is fitting the model with labelled data. In the case of water, this may require special purpose sensors, plumbing, and battery-operated equipment to be installed. Unfortunately, in real houses, it is not viable to install a flow switch in every fixture or a Closed Circuit Television (CCTV) camera in every room just to fit the classification model because plumbing is expensive and invasive. Any viable approach should then comply with the principle of minimal installation requirements, and, further, any sensors or equipment installed should already be an off-the-shelf product with a high degree of acceptance among the general public. In summary, we can identify three requirements for instrumenting a house with sensors:

- High acceptance (design, shape, part of shopping trends, identification of a user need)
- Low cost to buy and install
- Minimal or zero maintenance

All the works described in this survey challenges the previous state-of-the-art against classification accuracy and are thus built on some hi-tech lab-level setup that requires continuous manual intervention to ensure a reliable collection and processing of water data and ground truth. To summarize:

Flow traces analysis [34] requires a data logger to be installed and then data should be manually collected every 14 days, the data collected are then manually analysed and added to a database. It seems a feasible solution to analyse a given period of time, but is not practical to perform online disaggregation. HydroSense [38] reaches an accuracy of 80%, but needs at least two days of ground truth collection. Their current approach trains the language model using data from the home where it is deployed, but it is still an open issue as to how to leverage usage patterns across different homes to reduce the calibration phase of algorithms. The work in NAWMS [40] seems to be tested on only a lab testbed without any real world assessment. However, the use of low-energy wireless sensors deployed in a house seems promising. In Reference [30], the authors proposed a setup based on acoustic sensors on board of a wireless kit that is claimed to last longer than one year.

The summary above raises the following research questions: How likely are models fitted with labelled data from a training house “predictive” in another house? Or, in general, how can we assess the significance of a model trained on a subset of ground truth instrumented homes with respect to the general population of homes in a city or district? For each home, there are so many different variables, such as sizes, pipes lengths, fixtures, appliances, demographics, *etc.*, thus, we should consider the hypothesis that each house is a phenomena modelled by a set of completely different parameters. This diversity may narrow the applicability of disaggregation based on supervised machine learning because it is not feasible to instrument a massive number of houses with the necessary ground truth to build a training set, taking into account all the possible independent variables.

### 5.1. Acceptance of Smart Metering and Cognitive Metering

The topic of smart metering is not new and has already triggered many discussions and criticisms thus far. There are at least three types of issues identified by smart metering detractors. The first is in respect to health. Many movements and local communities [53] express concerns about the installation of a plethora of new wireless devices at home, which may cause an unexpected and unnecessary amount of radio pollution, which is, in turn, is blamed as a potential cancer cause. The second one is with respect to violations of citizens’ rights. Detractors accuse governments of being driven by the interest of suppliers and that the smart metering roadmap has been laid out without public consultation, in violation of the spirit of shared consensus and democracy [54]. The last concern, and probably the one with a proven impact, is that of privacy. There are many examples of how high-resolution metering could be used to identify personal habits and retrieve personal information. Notable proof of this concept is shown in Reference [55], where TV programs actually watched by home occupants is inferred by correlating features such as the luminosity of scenes to high-resolution energy consumption data. An approach to increase acceptance of industrial-level smart and cognitive meters a viable solution is twofold:

- Give control to end users (they must be able to switch on/off the metering; to set up the resolution; to control the amount of radio messaging inside the property, *etc*.)
- Locally process most of the data and locally reveal the insights needed by end users to monitor and improve their water demand. Powerful insight, such as usage disaggregation, could occur in-home rather than being inferred remotely. This allows to send, to the supplier, only the strictly-necessary data for operation (for instance daily average consumption over a week).

However, the above-mentioned approach does not take into full account the detailed needs of water suppliers, as the focus is mainly on user privacy. Thus, as explained in Reference [56], developing a context-specific framework for assessing how the collection and processing of detailed water usage impacts the user’s privacy, and identifying a set of best practices to mitigate the impact is of paramount importance. We expect that this issue will be addressed as soon as smart metering and cognitive metering become ubiquitously available for the adaptive management of urban water resources.

### 5.2. A Few Promising Research Directions towards Real World Adoption

In this section, some promising directions of further investigation are described. The general rationale is not to encourage competition in classification techniques to achieve 100% accuracy, but rather to bridge the gaps for water disaggregation to become a viable tool in real world environments.

#### 5.2.1. Pattern Mining and Unsupervised Approaches

In supervised approaches, building labelled data sets usually involves invasive lab-level equipment to be designed *ad hoc*, and manufactured for that purpose. If the problem is fitting models with labelled data, then the immediate alternative is using unsupervised learning techniques. In this respect, an unsupervised approach could be validated in a few houses and then scaled; in this field, some recent works have been proposed in the field of energy disaggregation [57], and it is likely that these may apply to the water domain as well. If we relax the goal of disaggregating load at the fixture level and with the highest accuracy, we can also consider the approach of detecting water signatures, as in Reference [58], where the objective was to define and identify a set of patterns (e.g., low but continuous flow over 48 h or more), which are quantitative and disaggregated assessment of water usages without precisely detecting which human activity is behind what usage. Bridging the gap between the aforementioned water signatures and the underlying human activities is a promising direction to investigate.

#### 5.2.2. Data Fusion

In general, the classification of fused data can yield better results than the classification over single data sources [59]. A promising direction of investigation is given by the nexus between energy consumption, water consumption, and human presence in a house (also gas metering could be an additional data source). In an extreme example, a 50% classification between laundry and gardening could be better disambiguated by the analysis of instant energy consumption given that one of the two activities uses energy and water at the same time. An example of the nexus between energy and water is presented in Reference [60]. In that paper, the authors leverage electricity non-intrusive load monitoring (NILM) to acquire water disaggregation as a set of water/energy correlated states.

#### 5.2.3. Working at Scale

Applying standard rates derived from sample studies is misleading because of the high variability in water use from one customer to another, even among customers with a similar infrastructure and social-economic profile. The model extracted from a single house’s data is limited, and does not leverage the information hidden in the broader population. What we consider parameters for a single house (pipe size, extension of parcel, number of rooms, habits of tenants, *etc*.) could be considered as independent variables in a broader model, comprising a full set of properties in a city or region. The collection of massive datasets for an entire city or region is, nowadays, technically feasible and possible to maintain in the long term. Hence, an interesting research question is to build and evaluate large-scale models.

## 6. Conclusions

Non-intrusive water disaggregation is a valuable approach for estimating fixture-specific water consumption, while keeping installation costs affordable, and, at the same time, the underlying complexity of processing remains manageable. We have presented a review of water disaggregation methods that make use of either mono-modal sensing or multi-modal sensing (e.g., combining different variables, such as water flow, pressure, *etc*.). The result of our review can be summarized in the following conclusions:

The field of water usage disaggregation is especially important in achieving water efficiency savings in domestic properties. This enables consumers to see data relating to which of their appliances utilize the most water, which appliances they use the most, and when they utilize appliances across the day. The availability of this subsequently enables a more efficient optimization of the consumer’s water usage by enabling them to reschedule usages at different times.

Generally, the setup to gather the training dataset for the supervised water disaggregation algorithm is expensive and unpractical in real world scenarios. Therefore, our opinion is that research should focus on unsupervised or semi-supervised learning methods. This would, not only make water disaggregation affordable and easy to deploy, but would also benefit from a wider acceptance from end users.

The concept of multi-modal sensing can further be exploited by applying data fusion techniques between water observations and energy observations. The water energy nexus needs to be formulated with a clear statistical hypothesis and, thus, assessed.

## Figures and Tables

**Table 1:** Average flow and average volume for the most common water usages and water events.

Usage/Event	Average Flow (L/min)	Average Volume (L)
Toilet flush	1–10	9–16
Shower	6–19	-
Dishwasher	5–7	15–40
Washing machine	-	45–170
Faucet (general usage)	7	-
Hand washing		5
Tooth brushing no saving		20
Tooth brushing water saving		1.5
Manual dish washing		40
Car washing		400
Faucet dripping	5 lt per day	
Irrigation	30–70	
Bath	19–30	70–170

**Table 2:** Indoor household use by fixture based on a large study involving more than 23,000 homes in North America.

Usage	Volume%
Toilet flush	24%
Shower	20%
Dishwasher	2%
Washing machine	16%
Faucet (general usage)	20%
Bath	3%
Leak	13%
Other	2%

**Table 3:** Summary of approaches and their characteristics in terms of output, resilience and installation.

Work	Approach	Installation	Output	Resilience
FlowTrace [34], Fondebaca *et al.* [36]	Water Flow	on top of regular water meter plus flow switches for ground truth	event type (accuracy 70%–88%) and volume (error ~30%)	issues in overlapping events
NAWMS [40]	Water flow + acceleration	smart meter on main supply, accelerometer per sub-pipe	flow rate estimation (error <10%)	suffers noise (external vibration)
Watersense [41]	Water flow + PIR	flow meter at house supply and motion sensors in each room	water flow (error 10%–20%) and fixture identification (accuracy 80%)	fails if 2 fixture same type used simultaneously
Ranjan *et al.* [32]	Water flow + RFID	flow meters at each fixture, two RFID readers in each door way, 15 RFID readers at fixture level.	event type and user mapping	issues if more users in proximity
Fogarty *et al.* [30]	Acoustic	4 sensors: 1 on cold water pipe, 1 on hot water pipe from heater, 2 on wastewater	event type (accuracy >90%)	works for overlapping events but with decreased accuracy
HydroSense [37,38]	Pressure	any accessible location under pressure	event type (accuracy 95%), fixture identity (accuracy >90%) & volume (error 5% -22%)	-
